# Supplementary figures and images for: A framework for mutational signature analysis based on DNA shape parameters
Source: PLoS One. 2022 Jan 11;17(1):e0262495. doi: 10.1371/journal.pone.0262495 (PMC8752002; doi:10.1371/journal.pone.0262495)

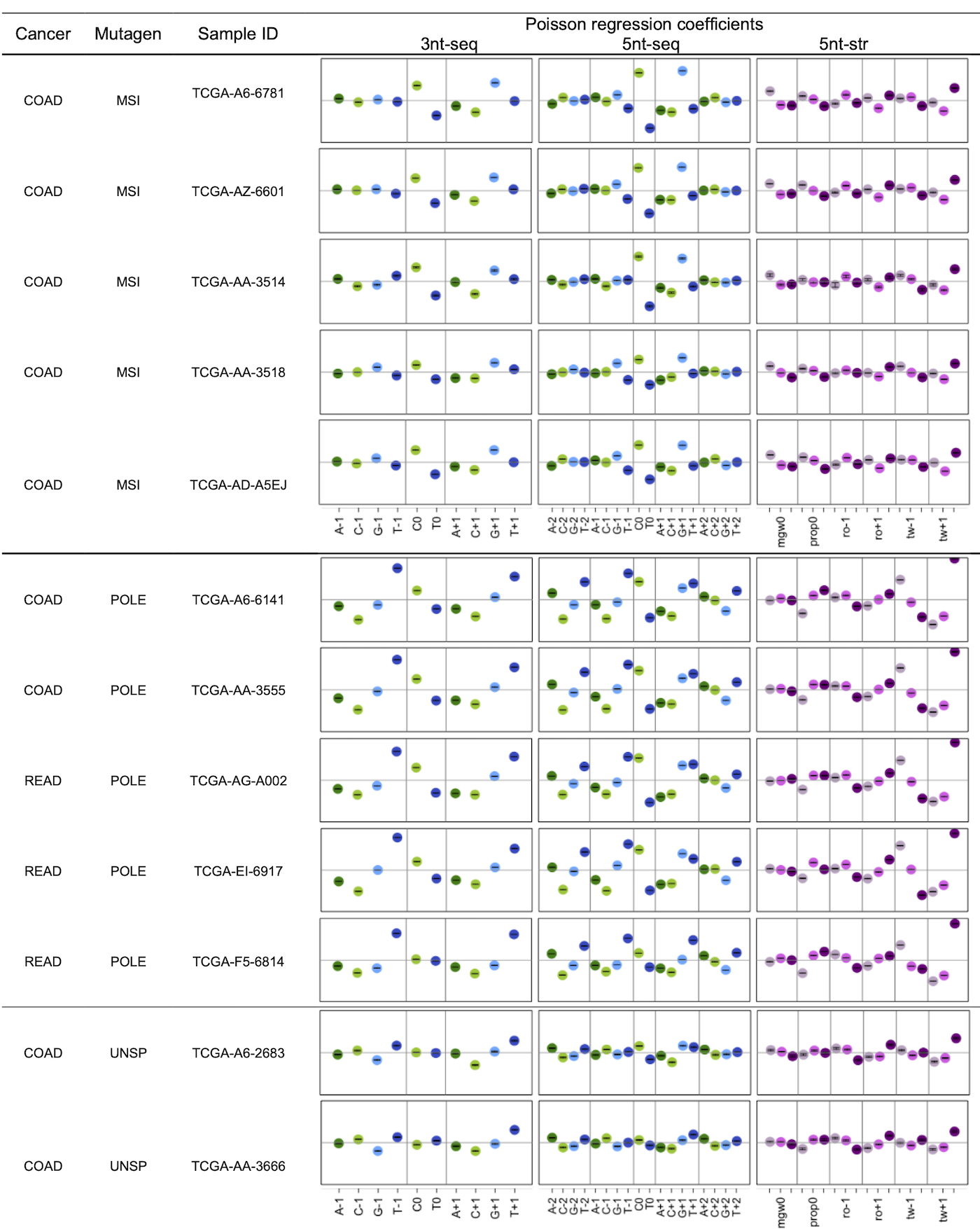

Supplement: S1 Fig — For the sequence block, the four DNA bases A, C, G, or T are color coded. For the structural block, parameters are normalized and divided into 3 equally spaced bins: high (light pink), medium (medium), and low (dark). Error bars superimposed on each symbol show 95% C.I. (TIFF) [file pone.0262495.s001.tiff]

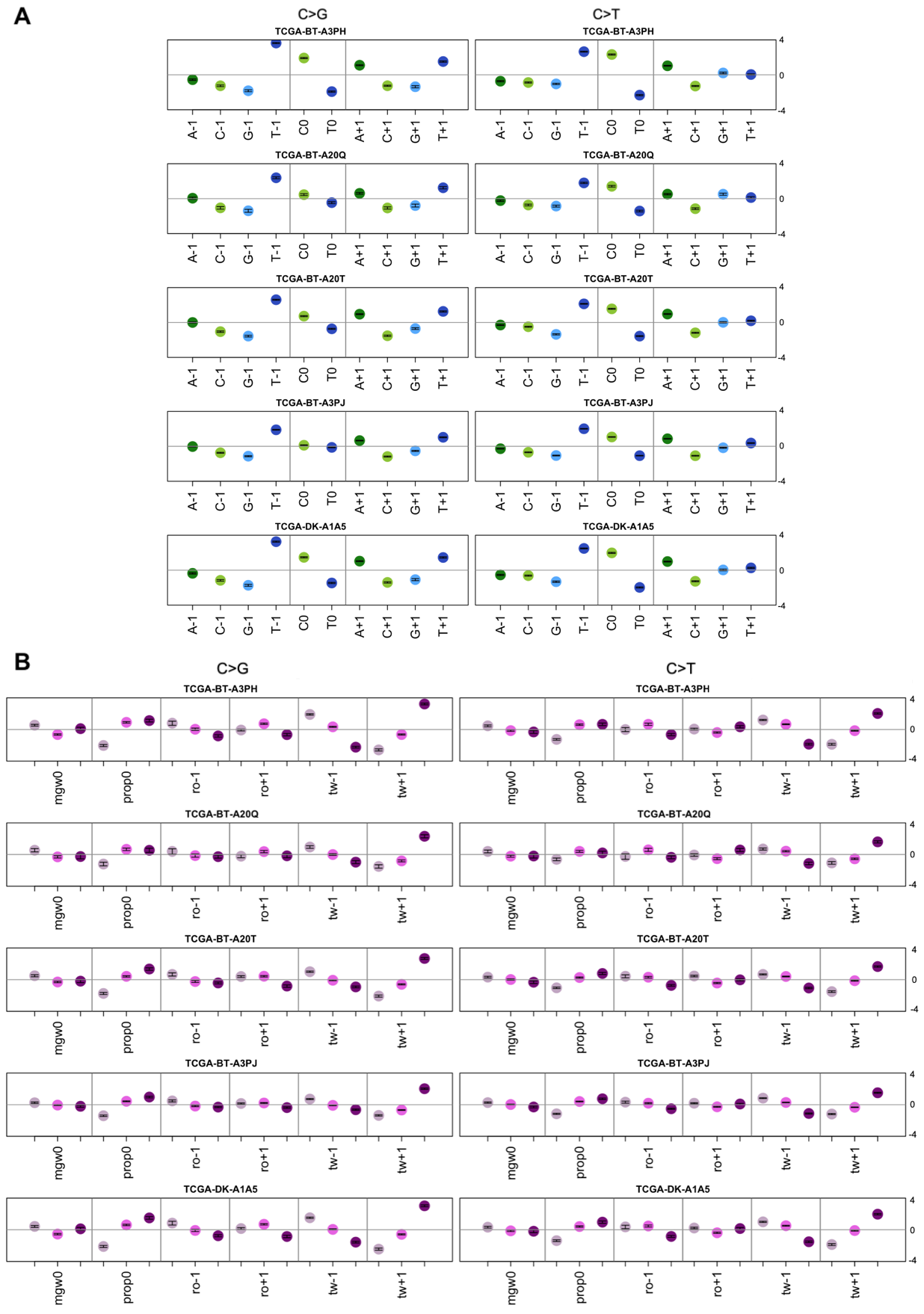

Supplement: S2 Fig — Shown for the (A) 3nt-seq and (B) 5nt-str models. (TIFF) [file pone.0262495.s002.tiff]

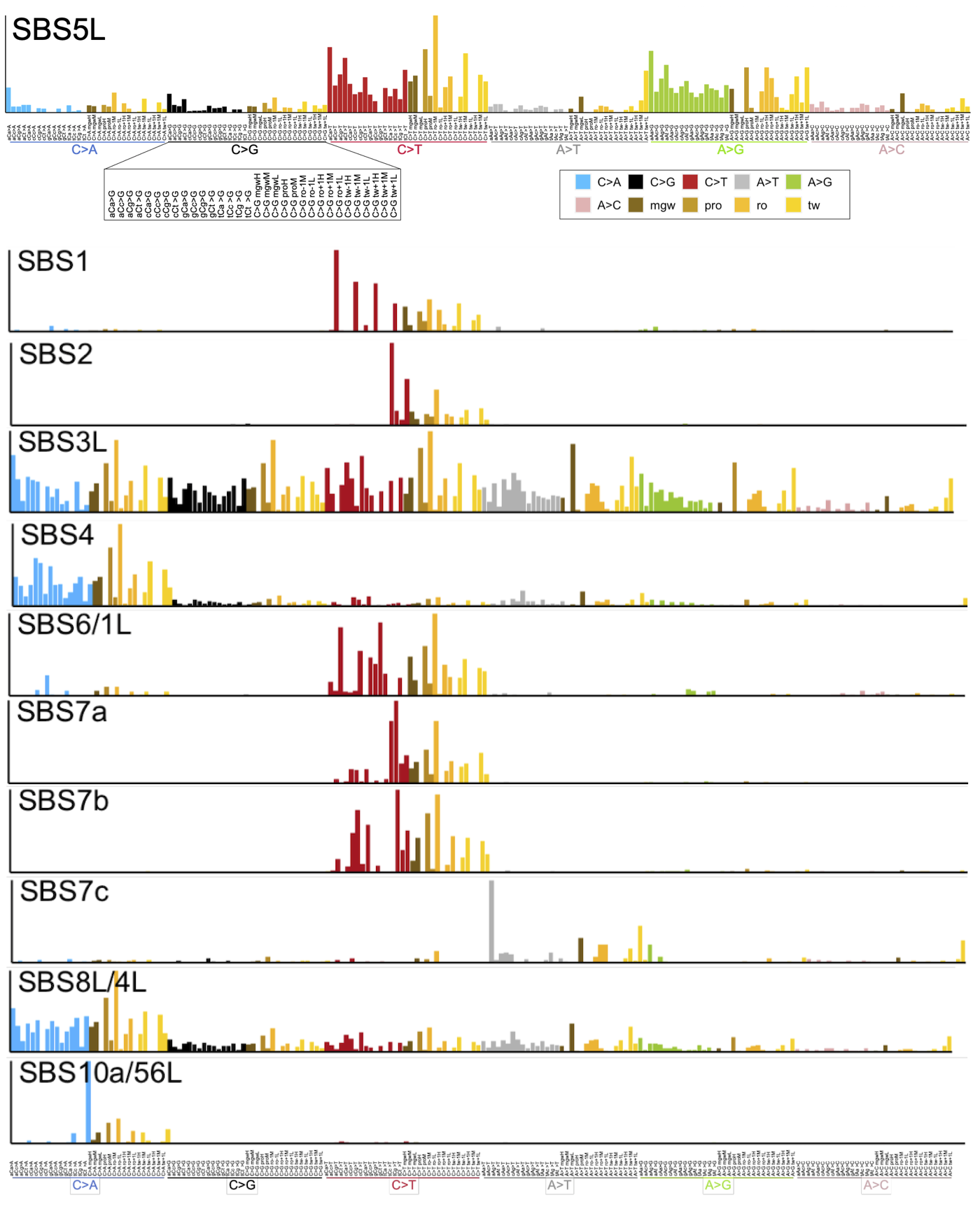

Supplement: S3 Fig — The composite mutational signatures consist of 96 DNA structural features: mgw and propeller at the central nucleotide (dark brown and light brown bars, respectively), roll and twist at -1 and +1 sites (dark yellow and light yellow bars, respectively), and the standard 96-component trinucleotide spectrum (blue, black, red, grey, green, pink bar colors). (TIFF) [file pone.0262495.s003.tiff]

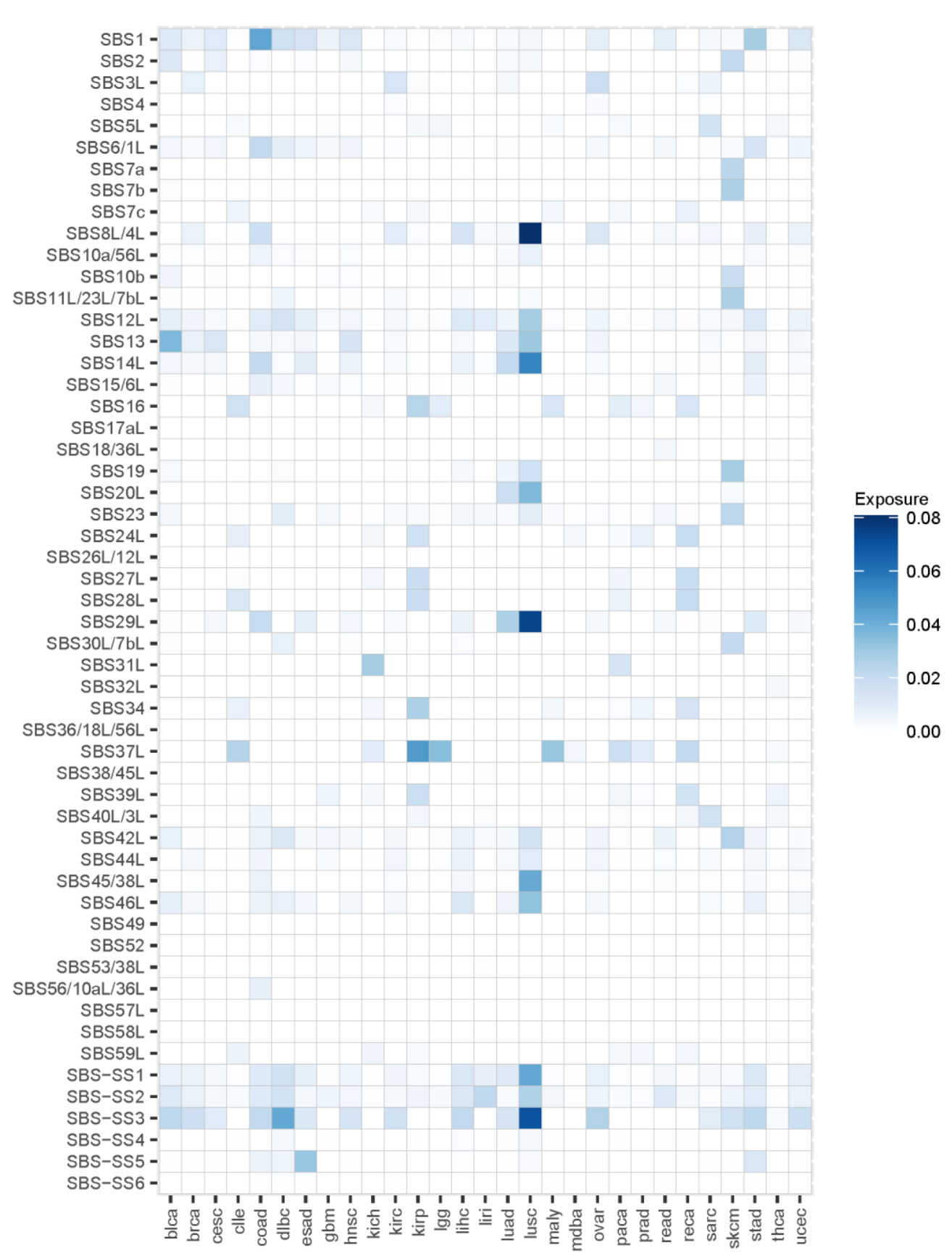

Supplement: S4 Fig — The mean exposure of all tumor samples in that cancer type is shown. (TIFF) [file pone.0262495.s004.tiff]

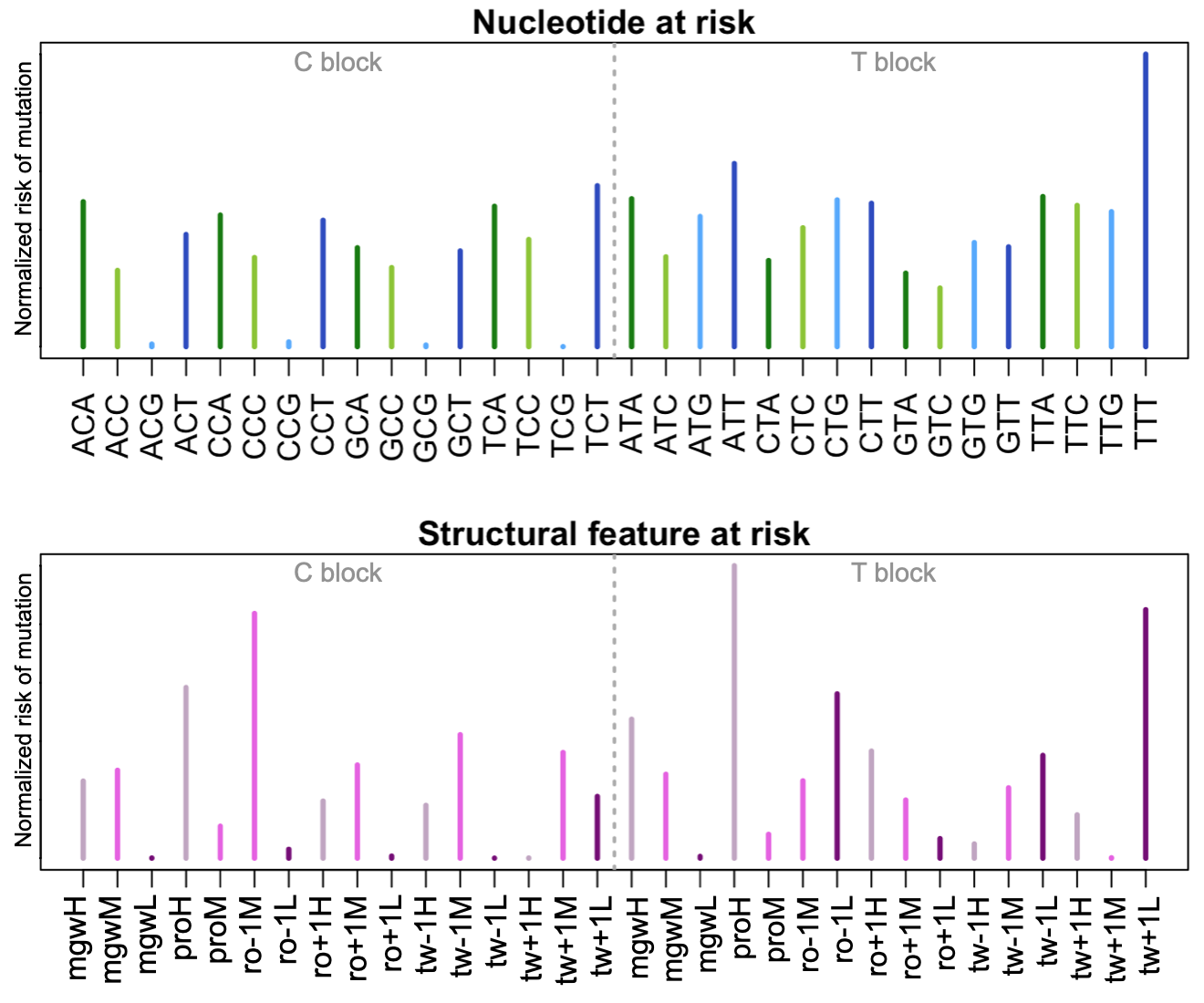

Supplement: S5 Fig — Number of nucleotides-at-risk in the human genome that may be affected by mutations, stratified by trinucleotide context (A) or by structural features (B). (TIFF) [file pone.0262495.s005.tiff]
