# Supplementary material for: A framework for mutational signature analysis based on DNA shape parameters
Source: PLoS One. 2022 Jan 11;17(1):e0262495. doi: 10.1371/journal.pone.0262495 (PMC8752002; doi:10.1371/journal.pone.0262495)
Supplement: S1 Table — (DOCX) [file pone.0262495.s006.docx]

**S1 Table. Fit of the Poisson regression models for additional examples of tumors affected by the mutagenic processes studied.** Fit (in columns 3nt-seq, 5nt-seq, 5nt-str and 7nt-str) was estimated as the McFadden pseudo-R^2^ statistic.

| Cancer | Mutagen | 3nt-seq | 5nt-seq | 5nt-str | 7nt-str | Sample ID | POLE Hotspot |
| --- | --- | --- | --- | --- | --- | --- | --- |
| COAD | MSI | 0.57 | 0.56 | 0.55 | 0.60 | **TCGA-A6-6781**^a^ |  |
| COAD | MSI | 0.70 | 0.56 | 0.56 | 0.58 | TCGA-AZ-6601 |  |
| COAD | MSI | 0.79 | 0.64 | 0.58 | 0.45 | TCGA-AA-3514 |  |
| COAD | MSI | 0.65 | 0.46 | 0.46 | 0.47 | TCGA-AA-3518 |  |
| COAD | MSI | 0.39 | 0.46 | 0.53 | 0.56 | TCGA-AD-A5EJ |  |
| COAD | POLE | 0.86 | 0.81 | 0.76 | 0.81 | **TCGA-A6-6141** | S297F |
| COAD | POLE | 0.86 | 0.81 | 0.76 | 0.79 | TCGA-AA-3555 | P286H |
| READ | POLE | 0.89 | 0.84 | 0.75 | 0.83 | TCGA-AG-A002 | S459F |
| READ | POLE | 0.84 | 0.77 | 0.70 | 0.79 | TCGA-EI-6917 | V411L |
| READ | POLE | 0.85 | 0.79 | 0.72 | 0.80 | TCGA-F5-6814 | P286R |
| COAD | UNSP^b^ | 0.43 | 0.25 | 0.38 | 0.37 | TCGA-A6-2683 |  |
| COAD | UNSP | 0.44 | 0.32 | 0.45 | 0.37 | TCGA-AA-3666 |  |
| BLCA | APOBEC | 0.94 | 0.92 | 0.77 | 0.85 | **TCGA-BT-A3PH** |  |
| BLCA | APOBEC | 0.86 | 0.80 | 0.68 | 0.59 | TCGA-BT-A20Q |  |
| BLCA | APOBEC | 0.89 | 0.86 | 0.72 | 0.79 | TCGA-BT-A20T |  |
| BLCA | APOBEC | 0.85 | 0.82 | 0.69 | 0.80 | TCGA-BT-A3PJ |  |
| BLCA | APOBEC | 0.92 | 0.90 | 0.75 | 0.83 | TCGA-DK-A1A5 |  |
| BLCA | UNSP | 0.78 | 0.65 | 0.61 | 0.44 | TCGA-BL-A13J |  |
| BLCA | UNSP | 0.80 | 0.64 | 0.58 | 0.39 | TCGA-BT-A20V |  |
| LUAD | SMOKING | 0.98 | 0.83 | 0.85 | 0.50 | **TCGA-44-2659** |  |
| LUAD | SMOKING | 0.95 | 0.86 | 0.78 | 0.62 | TCGA-50-5066 |  |
| LUAD | SMOKING | 0.94 | 0.80 | 0.75 | 0.70 | TCGA-55-7281 |  |
| LUAD | SMOKING | 0.81 | 0.56 | 0.55 | 0.71 | TCGA-64-1678 |  |
| LUAD | SMOKING | 0.97 | 0.92 | 0.84 | 0.59 | TCGA-73-4659 |  |
| LUAD | UNSP | 0.72 | 0.49 | 0.48 | 0.21 | TCGA-05-5429 |  |
| LUAD | UNSP | 0.65 | 0.31 | 0.35 | 0.11 | TCGA-44-2666 |  |
| SKCM | UV | 0.89 | 0.82 | 0.66 | 0.74 | **TCGA-D9-A148** |  |
| SKCM | UV | 0.90 | 0.83 | 0.69 | 0.76 | TCGA-ER-A19E |  |
| SKCM | UV | 0.89 | 0.84 | 0.66 | 0.79 | TCGA-FS-A1ZK |  |
| SKCM | UV | 0.90 | 0.84 | 0.68 | 0.80 | TCGA-EB-A24D |  |
| SKCM | UV | 0.85 | 0.80 | 0.61 | 0.78 | TCGA-EE-A2M5 |  |
| SKCM | UNSP | 0.68 | 0.34 | 0.42 | 0.16 | TCGA-ER-A19T |  |
| SKCM | UNSP | 0.68 | 0.38 | 0.40 | 0.17 | TCGA-FS-A1ZU |  |
| GBM | TMZ | 0.78 | 0.58 | 0.52 | 0.28 | **TCGA-14-1402** |  |
| GBM | TMZ | 0.76 | 0.57 | 0.51 | 0.30 | TCGA-06-0171 |  |
| GBM | TMZ | 0.82 | 0.66 | 0.59 | 0.37 | TCGA-06-0190 |  |
| GBM | UNSP | 0.66 | 0.35 | 0.32 | 0.09 | TCGA-02-2483 |  |
| GBM | UNSP | 0.64 | 0.22 | 0.25 | 0.09 | TCGA-06-0128 |  |

^a^ representative sample discussed in the main text shown in bold

^b^ unspecified mechanism(s)
