# Supplementary material for: A framework for mutational signature analysis based on DNA shape parameters
Source: PLoS One. 2022 Jan 11;17(1):e0262495. doi: 10.1371/journal.pone.0262495 (PMC8752002; doi:10.1371/journal.pone.0262495)
Supplement: Attachment — Submitted filename: plosONE_revisions_DNAframework_update_woNewFigs.pdf [file pone.0262495.s010.pdf]

**Responses to reviewers (Karolak et al., PLOS One)**

**Reviewer #1:**

Mutational signatures represent different mutational processes underlying cancer initiation and development, and they are usually reported by the frequency of tri/penta-nucleotide contexts. This study considers structural features that may enhance the interpretation of sequence-based signature. Overall, this work is of high quality. Although the ability of shape parameters to predict mutation probability is not better than sequence context, the incorporation of trinucleotide-based mutation type with structural-based parameters could provide new insights for mechanisms of mutagenesis.

> **Response to the Reviewer #1:** We thank Reviewer for the thoughtful feedback and an overall positive appraisal of our manuscript in terms of quality and potential to provide new insight. Their suggestions have helped improve our manuscript considerably.

**Reviewer's comments:**
**1**, The author should explain why only focus on these three shape parameters. They mentioned the effect of hairpin structure on mutation formation, but not actually include it as a parameter.

> **Response:** We agree that, in principle, a broader set of DNA shape parameters (e.g., the commonly used set of 13 parameters: minor groove width [mgw], 6 base pair step parameters [twist, roll, tilt, rise, slide, shift], and 6 base pair parameters [opening, propeller, buckle, stagger, stretch, shear]) could serve as predicting features for DNA mutability studies.
>
> However prior work has shown that the 4 of these – as used in our study: mgw, twist, roll, and propeller – are the more important structural features for a conceptually related problem: evaluating protein-DNA recognition and interactions (Rohs et al. Nature 2009; Gordan et al. Cell Reports 2013; Mathelier et al. Cell Systems 2016; Li et al. Nucleic Acids Research 2017). Thus, we focused on this more focussed, and less redundant set of features. There were additional reasons for reducing the set of DNA structure features: firstly, to match the number of features on the sequence context side, and secondly, to reduce the number of free parameters for regression models (which could lead to overfitting). We have now added the corresponding text and citations to the manuscript within the paragraph: "*DNA representation using structural features*" before the sentence: "*An illustration of the relationship* […]".
>
> Secondly, we thank the Reviewer for highlighting the relationship of DNA hairpin structure on mutation formation. This is known to be relevant for APOBEC-mutagenized cancer samples, since APOBEC3A can act on single stranded (ss) DNA loop within the hairpin [Buisson et al. 2019 *Science*]). Although we recognize that DNA hairpins are important structural forms for this type of DNA damage, the DNA structural parameters used in our work (and many prior studies, e.g., see above) are ill-defined inside the ss DNA segments. Thus, we think the issue of hairpins probably cannot be addressed fully within our current framework, which is adapted for double stranded (ds) DNA shape. An additional complicating issue is that the structures reported to be mutagenized in Buisson et al. are ~20 nt in length, considerably longer than the motifs within the scope of the current framework. Overall, we see it as an important direction for future work to be able to incorporate features relevant to ss DNA, as well as to adequately describe longer DNA motifs to predict mutation rates. We have now modified our manuscript, adding a statement to the Discussion to make clearer that the hairpins are not addressed by the current structural analysis, and that this and similar motifs would require additional methods.
>
> **We added the following text to Methods:** "Although all shape parameters including mgw, 6 base pair parameters, and 6 base pair step parameters could in principle generate predictive features for mutation risk, four of these (mgw, twist, roll, and propeller) were previously considered more relevant for evaluating protein-DNA interactions (Rohs Nature 2009; Gordan Cell Reports 2013; Mathelier Cell Systems 2016; Li

Nucleic Acids Research 2017) and thus by analogy we focus on these features also for mutation risk prediction. Additionally, using a more focused set of DNA structure features allowed us to match the number of features on the sequence-context side, and to reduce the numbers of free parameters in the signature analyses."

**We added the following text to Discussion:** "One caveat of the set of structural features currently employed is that their intended use is for ds DNA, however they are ill-defined in ss DNA segments. This means that the framework presented here, in its current implementation, would not be able to adequately address those mutational processes mediated by structured ss DNA. One known example are the hypermutable hairpin structures in DNA, where the loop is a target of the APOBEC3A cytosine deaminase (Buisson et al Science 2019) and is thus hypermutable (we note that only a minority of APOBEC3A mutations genome-wide is mediated by these stem-loop structures (Mas-Ponte Nature Genetics 2020)). Future work building upon our study may incorporate DNA structural descriptors pertinent to ss DNA, as well as increase the DNA oligomer length by drawing upon the ever-increasing amounts of cancer genomic data to increase statistical power."

These are the references cited:


- Li et al. Expanding the repertoire of DNA shape features for genome-scale studies of transcription factor binding *Nucleic Acids Res*. 2017, 45(22)
- Mathelier et al. DNA Shape Features Improve Transcription Factor Binding Site Predictions In Vivo, *Cell Systems*, 2016, 3(3)
- Gordân et.al. Genomic regions flanking E-box binding sites influence DNA binding specificity of bHLH transcription factors through DNA shape. *Cell Rep.* 2013; 3
- Rohs et al. The role of DNA shape in protein-DNA recognition. *Nature*. 2009; 461
- Mas-Ponte & Supek. DNA mismatch repair promotes APOBEC3-mediated diffuse hypermutation in human cancers 2020. Nature Genetics.
- Buisson et al. Passenger hotspot mutations in cancer driven by APOBEC3A and mesoscale genomic features. *Science* 2019.


**2**, For Fig2 and Fig3, the author only used one sample to represent the typical mutational processes. It would be good to see if the trend still holds for additional individuals as there are a lot of such samples available from TCGA/ICGC. In addition, POLE mutants have different hotspots (like V411L and P286R) with differential spectrum (Fang et al, 2020, Plos Gene; Hodel et al, 2020, Mol Cell), the sample used in this study should be indicated.

**Response:** We thank the Reviewer for a suggestion to support our findings with genomes from additional tumor samples.  We previously considered all 1637 WGS samples and selected only one hypemutated representative for each of the six representative mutational processes.

However, we do indeed agree that it is interesting to examine multiple tumor samples affected by the same process in order to check consistency of associations we reported between DNA structural features and mutagenesis by that process.

The tables below show analyses of the original representative tumor sample (n=6 total) and several additional samples (n=32), for a total of 38 tumor WGS analyzed by the Poisson regression.

Overall, these additional samples are remarkably consistent with the original sample in terms of overall model fit (as McFadden pseudo-$R^2$; Supplementary Table 1), and perhaps more interestingly, associations with structural features (regression coefficient profiles; Supplementary Fig. 1).

Related with this point: for the tissues considered (colon/rectum, bladder, lung adenocarcinoma, skin melanoma, glioblastoma) we have now included two samples per tissue (total n=10 out of 32) that we know do not exhibit the same dominant mutational process. This resulted in lower pseudo-$R^2$ measures for these samples (Supplementary Table S1) and changes in the regression coefficients (Supplementary Fig. S1) in those samples. This is consistent with the DNA structural features associating strongly with each single mutational process (as in the hypermutators), but not a nondescript mix of mutational processes.

Additionally, we also indicate the known POLE hotspot identified for the POLE samples (as mentioned in Shinbrot et al, 2014, Genome Research; Fang et al, 2020, Plos Genetics). The one POLE sample we originally studied had a less common S297F mutation and mutations of the remaining POLE samples are listed in the Supplementary Table S1.

The changes to the manuscript are listed below:

- Page 9 (now 11): To indicate the POLE sample we changed:
  "(ii) activity of the proofreading-deficient DNA polymerase epsilon (POLE) in another colorectal cancer sample" to: "(ii) activity of the proofreading-deficient DNA polymerase epsilon (POLE) in another colorectal cancer sample bearing a S297F hotspot mutation".
- Page 10 (now 11): We changed "(Figure 2A, "3nt-seq")" to "(Fig 2A, S1 Fig, "3nt-seq")".
- Page 12 (now 13): We changed "($pR^2$; see Methods)" to "($pR^2$; see Methods) (Table 1, S1 Table)".
- We added text describing the analysis of multiple hypermutated samples and the consistency of results therein, as well as adding text
- We added this text to the Results:
  - On Page 10 (now 11): "To check consistency of results across individuals, we considered additional samples affected by the same mutational processes (S1 Table, S1 Figure; 4 additional tumors per process, except TMZ where there were 2 additional tumor samples available). As a negative control, we further considered tumor samples originating from matched tissues, but not affected by the single-process hypermutation (S1 Table, S1 Figure)."
  - On Page 17: "For each of the six types of hypermutators studied, we considered additional tumor genomes affected by the same mutational process. The outcomes of the analyses were consistent across individuals, in terms of the overall fit of regression models, both the structure-based and the sequence-based ones (S1 Table), with some variability noted across MSI samples. Furthermore, the statistical associations of individual structural features with mutation rates appeared overall consistent across tumors affected by the same hypermutation mechanism (S1 Fig). As a control, we considered tumor samples matched by tissue but not affected by the same hypermutation mechanism; instead, the mutations in these control samples likely originated from a mixture of similarly prevalent mutagenic mechanisms. As expected, the fit of the regression models – either sequence or structure-based one – was poorer on the control tumors (S1 Table). Additionally, these control samples did not exhibit the same patterns associations of structural features with mutation rates as the original hypermutator samples (S1 Fig)."
- We added an additional Table and Figure to the Supplementary material and updated the numbers of current supplementary material.

**3**, Poisson regression to evaluate sequence-based variables and structural-based variables for mutation probability would be good to be presented by an equation in the method.

**Response:** We agree; we have added the Poisson model equation and included it in the Methods section of the manuscript:

"Poisson regression is commonly used for modeling count data. The model is given by:

$$log\ \lambda_i = \beta_0 + \beta_1 x_{i1} + \ldots + \beta_p x_{ip}, \qquad (1)$$

where $\lambda_i$ is a countable response variable, modelled as the linear combination of the covariates corresponding to the $i^{th}$ observation of the predictor variable $x$. Similarly to linear and logistic regression, the covariates are fixed, and the regression coefficients $\beta = (\beta_0,\dots, \beta_p)$ are the model parameters to be estimated. The countable response variable $\lambda$ can be substituted with the ratio $\lambda/D$ of the count of the events (here mutations) and total number of opportunities $D$ that the event had to occur. This leads to an extra term – an offset $log\ D$ – added on the right-hand site of the equation and an adjustment of the regression coefficients."

**4**, In page 34, I think the second paragraph is out of scope of this paper. Maybe it is better to discuss how the shape parameters incorporated signature would benefit to personalized therapy.

**Response:** We agree. We have now edited the paragraph to make it more concise, omitting peripheral information, and thus better focused on the potential for incorporation of shape parameters for patient stratification using genomics:

"There is a growing awareness that analysis of somatic mutation data may be able to provide markers to guide personalized therapy for cancer patients. Current focus is on refining drug selection to match the underlying genetic profile of tumors, particularly in terms of driver changes.[58,59,60] However, because mutational signatures reflect ongoing genomic instability, which is common in tumors and recognized as a targetable vulnerability of cancer, mutational signatures could help better stratify patients for targeted therapies, e.g., immunotherapy for MMR-deficient tumors.[61,62] The mutational Signature SBS3 and also a pattern of deletions with microhomology signal failures in the homologous recombination repair (HRR) pathway, which is targetable by PARP inhibitors.[19,20] This principle may extend beyond MMR and HRR deficiencies: in an analysis of mutational signatures across cancer cell line panels screened for drug activity, many signatures were associated with drug response, suggesting that some of the signatures might constitute useful genomic markers in patients.[21] For example, given that the error-prone DNA polymerase eta (POLH protein) can cause resistance to treatment with cisplatin,[63] identification of its mutational signatures – previously via DNA sequence features[47] but possibly also by the affinities of this error-prone DNA polymerase(s) towards certain DNA shape features (Fig 5) – could inform treatment decisions. In summary, there is great promise for clinical use of genomic predictive markers in tumors, even though often the underlying mechanisms remain elusive.[60] Among such genomic markers, the utility of mutational processes in particular merits more attention."

**Reviewer #2:**

In this study Karolak et al. described how data on DNA structure can predict mutation probability. They firstly analyzed the role of DNA structure and nucleotide context in mutation probability independently and then tried to extend the idea of standard mutational signatures (based on trinucleotide mutational spectrum) by including information about structural features. The idea itself looks very exciting. My main concern about it is prediction of structural features from DNA sequence and very strong correspondence between contexts and structures at such a small scale. Mutagenic processes in investigated cancers are highly context-dependent, so by definition you will have structure-dependence as well if each context corresponds to very specific structure. It would be informative to understand how much different contexts vary in described structural parameters. It would also be interesting to see whether different contexts with similar structure can have similar changes in mutation probability. Probably it would be helpful to investigate more large-scale structures to see whether there are differences in mutability of particular context in different structures? Similar to example with APOBEC in stem loops.
For investigated cancers, models with structural features performed similarly well to contexts. It would be interesting to investigate whether this is the case for cancers without such high context-specificity.
The most interesting part of the paper is prediction of new signatures that could not be extracted based only on

nucleotide context. Although the method seems not to be ideal and the biological interpretation is quite limited, this work is a good start point in this direction.

**Response to the Reviewer:** We thank the Reviewer for the positive evaluation of our work in terms of the idea being exciting, and in terms of our work being a good starting point for development of structural signature methods and of their biological interpretation. For the latter point (interpretation), we certainly agree that our study provides results that are preliminary in nature however, we would also argue that extending this study further would be more opportune for future work, rather than a revision for PLOS One.

Additionally, we appreciate the comments on the interest in sequence-structure correspondence patterns, in examining larger-scale structures' role in mutagenesis, and stem-loop structures' role. Some of these points require non-trivial extensions of the methodology, e.g., the hypermutable ssDNA segments within stem-loops, which are not well described with the current dsDNA-based DNA structure descriptors. These are certainly important directions for future studies, which we now make note of in the Discussion (see the response to Reviewer 1 question 1 for details, and the text we added).

Overall, we think the reviewer's specific comments were constructive and helped improve our manuscript; our point-by-point responses are below. In addition, we would like to draw the reviewer's attention to the new Supplementary Table 1 and Supplementary Figure 1, where we show associations of structural features in additional examples of tumor genomes affected by the mutational processes we addressed earlier. This demonstrates that the associations reported are indeed robust across 'biological replicates' i.e., repetitions of the natural experiment that is tumor evolution.

**Comments:**
**1**. Figure 1 is not fully understandable. From the scheme, it seems that far right and far left nucleotides are not engaged in any parameter estimation that seems not to be true.

> **Response:** We thank the Reviewer for the comment on schematic in Figure 1; we see how this could be seen as confusing. We have now added the explanation why five nucleotides are needed to calculate minor groove width (mgw), base pair, and base pair step parameters to the caption of Figure 1. Although the flanking nucleotides appear unused in the calculations of the parameters, the whole pentamer DNA motif is necessary to calculate mgw and two of the base pair step parameters (Zhou et al, 2013) and the flanking nucleotides do enter the calculation this way.
>
> **Updated Figure 1 legend by appending**: "Considering the DNA pentamer sequence is an approach to derive DNA structural features at a locus, while accounting for the nearest and next-nearest neighbors of the central nucleotide. The structural features: mgw, the one bp, and the two bp step parameters, at a given central base pair are the function of its pentamer environment."

**2**. Is it correct that Poisson regressions do not take information about mutation type into account? I mean that coefficients on Figure 2 and Figure 3 show influence on mutation probability of central nucleotide independently of mutation type. In this case, they seem to be biased by the main mutation type in cancer.

> **Response:** Indeed, this should be clarified. The reviewer is correct in that the regression coefficients show association with mutation probability in the central position, while jointly considering all mutation types (e.g. in case of APOBEC mutagenesis that has both C>T and C>G in the spectrum, both the neighborhoods of C>T and C>G mutations are considered equally). We think the underlying assumption is reasonable: in the same mutagenic process, different mutation types are likely to originate from the same or at least a shared mechanism (e.g., APOBEC mutagenesis always starts by deamination of C to U, which can later either be repaired correctly [C>C] or it can resolve to either C>T or C>G mutations). Based on this assumption, the mutational events originating from one process can be treated as a set; this has the practical advantage of increasing statistical power to find associations due to larger sample sizes. To evaluate if this

assumption broadly holds, for the APOBEC example we checked the structural and sequence features associated with C>T mutations separately, and with C>G mutations separately, in the Poisson regression (see new S2 Fig). We observe broadly similar trends across the two APOBEC mutation types, C>T and C>G, suggesting this assumption is reasonable.

With respect to these regression coefficients being biased by the major mutation type in that cancer: we would not call it a bias. In determining the coefficients, regression will place a higher weight on the more common mutation type in that tumor, which we think is the sensible thing to do if the goal is to better represent all mutations in that tumor. We have now edited the the Methods section of the text to clarify how the mutation type is treated in the regression. This is the text we added:

"The mutation type is not explicitly considered in the Poisson regression as implemented, but instead all mutations occurring at a C:G pair are considered jointly (C>A, C>T and C>G), and all mutations considered at an A:T pair are considered jointly (T>A, T>C and T>G). The assumption underlying this is that in a tumor predominantly affected by a single mutagenic process, different mutation types are likely to originate from the same or a shared mechanism. This provides a rationale for treating various mutation types as a single set, thus increasing statistical power to find associations. Of note, the more commonly observed mutation types in a particular tumor sample will have a higher weight towards determining coefficients in the regression run on that tumor sample."

Finally, we would also like to note that the NMF analysis for mutation signature extraction does treat the structural features separately by mutation type. Indeed, if there were any differences in, for example taking the APOBEC mutagenesis, the structural features relevant for the C>G versus the C>T mutations, this would be evident in the signatures extracted by NMF.

**3**. Could you please discuss in text why 3nt-seq model always performs better than 5nt-seq model? (Table 1).

**Response:** We thank the Reviewer for this question. In fact, we had discussed this extensively while preparing our manuscript. We now have added a brief discussion of this to the Results section:

"Consistently across samples, we noticed that performance of the trinucleoide DNA sequence models (3nt-seq) outperforms performance of pentanucleotide sequence models (*5nt-seq*) (Table 1, Supplementary Table 1). This may be due to data that are sparser, i.e., many more of the mutation count values in the regression tables for *5nt-seq* are zero, due to pentanucleotide motifs being longer and an individual pentanucleotide occurring less frequently genome-wide compared to an individual trinucleotide."

**4**. Page 13 "the structural DNA features compared favorably (pR2=0.81 for 7nt-str, Table 1) to the sequence DNA features (pR2=0.81 for 5nt-seq)". Why do you call this comparison favorable if they have equal pR2?

**Response:** This comment has been addressed by amending the Results text, which now states:

"As with the MSI tumor, also in the POLE tumor the structural DNA features yielded a similarly well-fitting model ($pR^2$=0.81 for 7nt-str, Table 1) as the sequence DNA features did ($pR^2$=0.81 for 5nt-seq). This further supported that the structural features we examined are appropriate to describe the propensity of DNA sites to mutate in DNA repair-deficient tumors."

**5**. Page 13 "The overall predictive accuracy was higher for the POLE tumor than for MSI tumor (Table 1), suggesting that mutational risk can be predicted from DNA shape to a variable degree across different mutational processes." From Table 1 it is seen that context-model also performs better for PolE compared to MSI. As

structural features were predicted from nucleotide sequence this fact can probably be explained by lower context-specificity of MMR compared to PolE.

> **Response:** We understand the point the reviewer is making, and we agree. The statement as originally written did not imply that the context-model did not perform better, although we do see how the wording could be understood as such. To clarify, the Reviewer's interpretation is now made explicit in this text segment:
>
> "The overall predictive accuracy was higher for the POLE tumor than for MSI tumor (Table 1, S1 Table). This suggests that mutational risk can be predicted from DNA shape to a variable degree across different mutational processes, similarly so as when predicting mutational risk from DNA sequence."

**6**. Page 14 "Considered together with over- and under-twisting at positions -1 and +1, respectively, this suggests that DNA motifs experiencing APOBEC mutagenesis may be more prone to have flipped out bases". It is interesting to investigate the co-occurrence and simultaneous influence of structures found significant in Poisson regression.

> **Response:** We agree, it is indeed interesting to analyze the co-occurrence and simultaneous influence of structural features found significant through Poisson regression (PR). We discuss examples of this throughout the text including PR analyses of POLE sample (page 13), tobacco smoking sample (higher values for propeller and roll, page 15), or within the new signatures' discussion (SBS4 and SBS6/1L, page 22).
>
> For some of the shifts in structural feature values (e.g., over- and under-twisting of the adjacent bases) our current knowledge allows to understand the conformational changes in DNA taking place (e.g. flipped out bases, in this particular example). However, to interpret other co-occurrences of structural features, it would be necessary to have an analysis using molecular simulations (on longer time scales) to better understand structural changes taking place. This would be, in our opinion, out of scope for the current study, but is certainly an interesting focus for future work.

**7**. Figure 5. X-axis is impossible to read. Structural signatures seem to be very similar between different signatures in contrast to difference between context signatures. For example, structural elements for SBS1, SBS3L, SBS6, SBS7b seems very similar for C>T mutation but contexts differ extremely. Could you explain this? I lack the interpretation as I was not able to understand the correspondence of bars to structural features.

> **Response:** We thank the Reviewer for this comment. We have changed the two-column arrangement of Figure 5 to one-column to display individual signatures more clearly and to make each structural parameter easily recognizable. We also improved the legend within the figure.
>
> We agree with the reviewer's observations that the contributions of structural elements for some of the signatures appear similar (e.g., when looking at C>T changes), even though the contribution of the sequence trinucleotide contexts appears to be much different.
>
> The reason for this is quite simple: the baseline frequencies for trinucleotides (i.e., the number of times they are seen in the human genome) are quite uniform, except for the NCG dinucleotide which is rarer than others. However, when considering various combinations of structural features across the genome, their abundance is rather diverse: some combinations of structural features are quite rare. (Speculatively, reasons for that could be that they are disfavored by physics and/or by evolution, however this does also depend a lot on the implementation of the methodology, in particular the discretization thresholds used to make low/medium/high bins). Thus, for a certain combination of physical features, the baseline "nucleotides-at-

risk" may be much lower and for another higher, and this will be reflected in all mutational signatures. This is because NMF, in its usual setup for mutational signature analysis, operates on the raw (unnormalized) counts, and the raw counts are trivially correlated with the number of "nucleotides-at-risk" for each feature.

To address this question with data, we now include a chart with the "nucleotides-at-risk" counts for the trinucleotide spectra, and also separately for the loci stratified by the DNA structural features; this provides a baseline for interpreting the signatures. Indeed it can be observed some of the mutation contexts, as currently stratified by the structural features, are much less common than others, which can give an impression of high correlation between the structural signatures (we note that the signatures could also easily be normalized by this baseline, which we provide in a supplementary table).

We now include the following text in the MS to explain this matter:

"A further methdological consideration is that these NMF signatures were derived from raw mutation counts, thus their shapes in part reflect also the number of genomic nucleotides-at-risk encompassed by each feature in the spectrum (S5 Fig). This holds true both for the trinucleotide part of the spectrum (e.g. the NCG trinucleotides are rare in the human genome), and also for the structural features (e.g. *mgwL* bin, using the current definition of L, M and H thresholds, is rarer than *mgwM* and *mgwH* bins, S5 Fig). This may result in an apparent similarity of the profiles across different signatures."

**8**. Figure 6 looks confusing. For example, SBS4 that you describe as tobacco smoking induced was not found in any lung cancer. For LUSC you determined high exposure to SBS8L/4L that is also similar to SBS4 but for luad you didn't observe any of them. SBS7a,b,c are not pronounced in skcm.

**Response:** We agree with the reviewer that the former Figure 6 (referring to the 'exposures' of our mutational signatures) may be able to be improved in future work.

It was recognized in the field that inferring mutational signatures, as well as estimating their 'exposures' in genomes, can be difficult. For instance it is clear that signature SBS8 (that the reviewer mentions) is in analysis usually difficult to unmix from SBS5 and SBS3 (recently reviewed e.g. PMID 34316057).

We think that in our analysis the tobacco-smoking C>A rich signature SBS4 is likely mixed with a similar tobacco-chewing signature SBS29L which is also C>A rich. This latter signature we observe rather enriched in both LUSC and also LUAD, just as the reviewer notes should be the case for a tobacco-associated signature. We edited the text to acknowledge that we might be confounding the two tobacco signatures into SBS29L in our analysis; given their similarity we don't necessarily see it as critical issue at this point. We do acknowledge that ideally -- perhaps with more tumor samples analyzed – such related signatures may be successfully disentangled.

We added this text: "While many extracted mutational signatures closely resembled their Cosmic counterparts in the trinucleotide spectrum, we recognize that inference of some of known signatures may be able to be further improved. For example, the signature SBS29 was previously associated with tobacco chewing. In our analysis, SBS29L may be a mix of the two similar, C>A-rich signatures: the tobacco chewing SBS29, and the tobacco smoking signature SBS4 (judging by its exposure in two lung cancer types, S Figure)."

About the example of the SBS7 (UV-associated) signatures the referee mentions: we do not think there is a problem there – SBS7a and SBS7b are indeed very strongly enriched in skin overall, a large cohort of which was labelled 'mela' in our previous plot (previously we had a 'mela' and 'skcm' datasets separation which was not necessary, now these samples were merged under 'skcm' and the plot updated – now it is in S4 Fig).

**Misprint**
**1**. Page 19 “suggesting that both DNA sequence features and the and DNA structural features should be considered jointly…”

**Response:** We corrected this error: extra “the and” was removed.

**Misprint noticed by authors:**

Figure 4 caption: change (K-L) to (I-J).

~ end ~
